# Benzodiazepines versus non-benzodiazepine antiseizure medications as first-line agents for status epilepticus: analysis of real word data from a 9-years prospective cohort

**DOI:** 10.3389/fneur.2025.1681483

**Published:** 2025-11-12

**Authors:** Francesco Brigo, Gianni Turcato, Giada Giovannini, Simona Lattanzi, Arian Zaboli, Niccolò Orlandi, Margherita Burani, Lisa Taruffi, Leonardo Affronte, Stefano Meletti

**Affiliations:** 1 Innovation, Research and Teaching Service (SABES-ASDAA), Bolzano, Italy; 2 Department of Internal Medicine, Hospital of Santorso (AULSS-7), Santorso, Italy; 3 Neurophysiology Unit and Epilepsy Center, Azienda Ospedaliera-Universitaria di Modena, Modena, Italy; 4 Department of Experimental and Clinical Medicine, Neurological Clinic, Marche Polytechnic University, Ancona, Italy; 5 Department of Biomedical, Metabolic, and Neural Sciences, University of Modena and Reggio-Emilia, Modena, Italy

**Keywords:** benzodiazepines, mortality, prognosis, propensity score, status epilepticus

## Abstract

**Background and objectives:**

The treatment of status epilepticus (SE) follows a stepwise approach, with benzodiazepines (BDZ) being the first-line therapy. This study analyzed real-word data on use of BDZ and non-BDZ antiseizure medications (ASMs) in SE treatment over 9-years to evaluate whether non-BDZ given as a first-line treatment affect 30-day mortality and other outcomes.

**Methods:**

We included SE cases in patients aged ≥14 years who were prospectively registered at Baggiovara Civil Hospital (Modena, Italy) between September 1, 2013, and October 31, 2021. First-line treatment choices were dichotomized as: (i) i.v. BDZ; (ii) other ASMs. A multivariate model with logistic regression and an adjusted stepwise method for variables was used. Then, a propensity-score matched analysis was performed with clinical variables unevenly distributed between the two groups to evaluate the independent association between first-line therapy and 30-day mortality and secondary outcomes.

**Results:**

Six hundred and thirty patients were included: 73.5% (463/630) received a BDZ as first-line therapy and 26.5% (167/630) were treated with non-BDZ. In the primary analyses of the whole cohort, 30-day mortality was 25.9 and 35.3% in patients receiving BDZ and non-BDZ, respectively (*p* = 0.027). However, multivariate analysis adjusted for potential confounders showed that non-BDZ treatment was not independently associated with increased 30-day mortality. Patients who received BDZ as a first-line treatment had less orotracheal intubation and anesthetics within 24 h of SE onset; less frequent progression to refractory and super-refractory status epilepticus; less admission to and shorter stay in intensive care units; shorter time to SE cessation. In the propensity cohort (140 patients, mainly non-convulsive SE; NCSE), 30-day mortality was 30.7% (43/140), with no difference between BDZ-treated patients (30%; 21/70) and those who received non-BDZ (31.4%; 22/70) (*p* = 1.000). No difference in secondary outcomes was found, except for a shorter time to SE cessation among BDZ-treated patients.

**Conclusion:**

The use of non-BDZ first-line treatment was found to be frequent, approaching 25%. Our propensity-score matching analysis shows that in some patients, mainly with NCSE, the overall prognosis of SE was not affected by first-line use of non-BDZ drugs. In these cases, SE prognosis might only be partially dependent on the first medications administered and could be more influenced by other biological variables.

## Introduction

Status epilepticus represents a time-dependent neurological and medical emergency that needs to be promptly diagnosed and adequately treated to reduce the risk of morbidity and mortality, which is around 15% but can be as high as 30% in the elderly (1–3). If the epileptic activity continues, SE can become refractory or super-refractory to medications and anesthetics, with increased risk of negative long-term effects, including “neuronal death, neuronal injury, and alteration of neuronal networks, depending on the type and duration of seizures” (4, 5).

The treatment of SE usually follows a stepwise approach. As a first-line treatment for SE, clinical practice guidelines consistently recommend the use of benzodiazepines (BDZ), mostly intravenous (i.v.) diazepam or lorazepam, or intramuscular midazolam (6–8). These drugs exert their antiseizure properties by binding to gamma-aminobutyric acid (GABA)-A receptors, increasing channel opening frequency at the receptor and, as a result, chloride conductance and neuronal hyperpolarization, resulting in greater inhibitory neurotransmission and antiseizure effect (9, 10). The rationale for utilizing these medications as first-line therapy is based on their efficacy, which has been established in multiple randomized controlled trials (RCTs) (11–14), and on their rapid onset of action, which is regarded as a key precondition for attaining rapid SE cessation.

Despite the existence of established guidelines, there is evidence of underuse and suboptimal dosing of BDZs, highlighting significant variability in clinical practice (15–17). Moreover, the impact of deviations from guideline-recommended treatment on clinical outcomes in SE remains an ongoing topic of research and debate (18–21).

This study aimed to evaluate whether non-BDZ ASMs given as a first-line treatment for SE affect 30-day mortality and other prognostic outcomes, such as SE cessation, progression to refractory or super-refractory SE, need for anesthetics or orotracheal intubation, admission to intensive care unit, and functional outcomes. For this purpose, we used data prospectively collected over 9 years of clinical practice in the treatment of SE at the academic hospital of Modena, Italy.

## Methods

### Study design, setting, and patients

We conducted a retrospective analysis of consecutive occurrences of SE in patients aged ≥14 years at Baggiovara Civil Hospital (Modena, Italy) between September 1, 2013, and October 31, 2021. Patient data were collected prospectively at the time each patient was treated in the hospital during the study period. Prior to 2015, SE was defined as a continuous seizure lasting 5 min or more, or two or more separate seizures with no complete recovery of consciousness between them (22). After 2015, the International League Against Epilepsy (ILAE) definition was systematically adopted and prospectively applied (4). Accordingly, the operational time for diagnosing SE was set at 5 min for tonic–clonic SE, 10 min for focal SE with impaired consciousness, and 10–15 min for absence SE. Two of the authors (SM and GG) reviewed all cases of SE that occurred prior to 2015 to ensure that they met the ILAE diagnostic criteria. The non-convulsive SE cases were diagnosed using the Salzburg EEG criteria (23, 24). Regarding EEG utilization, patients underwent EEG monitoring based on clinical indications; however, continuous EEG was not consistently implemented across all cases. Importantly, EEG was essential for confirming the diagnosis of non-convulsive SE and was employed in cases of SE with persistent impairment of consciousness after treatment or in instances of super-refractory SE.

Patients who had been intubated outside of the hospital were excluded, as they often received BDZ or other drugs with antiseizure properties to facilitate intubation.

A specific data form was used to collect demographic and clinical information, such as age, gender, medical history and comorbid medical conditions, prior history of epilepsy, etiological ILAE classification (4), in which acute symptomatic causes were classified as hypoxic or nonhypoxic, impairment of consciousness prior to treatment, SE semiology, impaired consciousness before treatment. The form was completed prospectively by the patient’s initial physician (neurologist or neurointensivist). Every patient who had a suspicion or a diagnosis of SE was sent to a specialist neurologist for both diagnostic confirmation and therapy. Even in the intensive care units, the consultant neurologist and an EEG recording are available 24 h a day, 7 days a week.

The treatment was carried out in accordance with an internal protocol (Supplementary material) based on international criteria (6–8).

For this study first-line treatment choices were dichotomized as: (i) i.v. benzodiazepines; (ii) other ASMs (non-BDZ).

### Outcome

The primary outcome was 30-day mortality. We also analyzed the following secondary outcomes: need for anesthetics within 24 h of SE onset; progression to refractory SE; progression to super-refractory SE; need for orotracheal intubation; admission to intensive care unit; length of stay in intensive care unit (days); return to pre-SE clinical condition at discharge; SE cessation; time to SE cessation; worsening of functional status.

Cessation of SE was defined according to the sustained effort network for treatment of status epilepticus (SENSE) study as follows: cessation of SE within the first hour after treatment initiation for generalized convulsive SE; cessation of SE within 12 h after treatment initiation for other SE types (21). Worsening of functional status was defined as a modified Rankin scale (mRS) at discharge higher than pre-SE mRS values.

Outcome data were gathered from the SE data set used to collect information and confirmed through the registry office. Our hospital information system integrates data from rehabilitation facilities and the province’s death registry, showing the living/deceased status of patients, with the date of death. For the few patients included in the study but residing in other provinces or regions of Italy, patient status was verified through telephone calls.

### Statistical analysis

The categorical variables were described as a percentage and the total number of events, and univariate comparisons were made using the Fisher exact test or the *χ*^2^ test. Depending on the underlying distribution, continuous variables were given as median and interquartile range (IQR) or mean and standard deviation (SD). The Mann–Whitney or *t*-tests were used to make comparisons.

A multivariate model with logistic regression and an adjusted stepwise method for variables found significant in univariate analyses and included as possible multivariate confounders was used to investigate the possible independent association between first-line treatment and 30-day mortality. The log-rank test was used to compare the survival of patients treated with BDZ as first-line therapy to patients treated with a different ASMs.

Subsequently, considering that different characteristics could have influenced the initial treatment choice, the baseline variables that were found to be unbalanced between the two groups were incorporated in a propensity score matching. As a result, a sub-cohort of patients was produced with baseline data evenly distributed according to first-line treatment (BDZ versus a different medication).

The analyses were performed after propensity score matching to evaluate the independent association between first-line therapy and 30-day mortality and the secondary outcomes mentioned above. All tests were two-sided, with a *p*-value <0.050 being statistically significant. Stata version 16.0 (StataCorp) was used for statistical analysis.

### Standard protocol approvals, registrations, and patient consents

The study was approved by the local ethical committee (ethics committee approval number 556/2018/OSS/AOUMO-RF201602361365) and was carried out in accordance with the Declaration of Helsinki’s ethical guidelines for medical research involving human beings.

## Results

Out of 711 potentially eligible patients, 81 were excluded because they had been intubated outside of the hospital due to airways protection need. As a result, 630 patients were eventually included in the study (Table 1). 73.5% (463/630) of patients received a BDZ as first-line therapy, while the remaining 26.5% (167/630) were treated with non-BDZ ASMs (Table 2). Among patients who received a BDZ as first drug, 74.3% (344/463) were treated with diazepam, 12.3% (57/463) with lorazepam, 11% (51/460) with delorazepam, and 2.4% (11/463) with midazolam. Patients who received a BDZ as first-line treatment were less likely to have impaired consciousness before treatment, had a higher history of previous seizures and lower comorbid ischemic heart disease, a lower incidence of acute hypoxic SE, and a higher incidence of progressive symptomatic SE. They experienced a higher frequency of generalized convulsive and focal motor SE and a lower frequency of non-convulsive SE.

**Table 1:** Baseline characteristics of included patients.

Variable	Total (*n* = 630)	BDZ first-line (*n* = 463)	Non-BDZ first-line (*n* = 167)	*p*-value
Patients, *n* (%)	630 (100)	463 (73.5)	167 (26.5)	
Sex, *n* (%)				0.309
Male	245 (38.9)	186 (40.2)	59 (35.3)	
Female	385 (61.1)	277 (59.8)	108 (64.7)	
Age, years, median (IQR)	75 (63–82)	75 (61–82)	75 (65–83)	0.189
Etiological classification, *n* (%)				
Acute symptomatic, hypoxic	58 (9.2)	30 (6.5)	28 (16.8)	**<0.001**
Acute symptomatic, non-hypoxic	344 (54.6)	249 (53.8)	95 (56.9)	0.526
Remote symptomatic	107 (17)	83 (17.9)	24 (14.5)	0.337
Progressive symptomatic	97 (15.4)	80 (17.3)	17 (10.2)	**0.033**
Other	10 (1.6)	21 (4.5)	3 (1.8)	0.156
SE semiology, *n* (%)				
Generalized convulsive	91 (14.4)	80 (17.3)	11 (6.6)	**<0.001**
Focal motor	169 (26.8)	148 (32)	21 (12.6)	**<0.001**
Non convulsive	345 (54.8)	221 (47.7)	124 (74.3)	**<0.001**
Myoclonic	25 (4)	14 (3)	11 (6.6)	0.061
Prior history of epilepsy, *n* (%)	223 (35.4)	179 (38.7)	44 (26.3)	**0.005**
Impaired consciousness before treatment, *n* (%)	199 (31.6)	119 (25.7)	80 (47.9)	**<0.001**
Comorbidities, *n* (%)				
Ischemic heart disease	76 (12.1)	48 (10.4)	28 (16.8)	**0.037**
Cerebrovascular disease	107 (17)	74 (16)	33 (19.8)	0.280
Diabetes mellitus	127 (20.2)	88 (19)	39 (23.4)	0.260
Chronic heart failure	44 (7)	30 (6.5)	14 (8.4)	0.478
Dementia	110 (17.5)	79 (17.1)	31 (18.6)	0.721
Tumor	61 (9.7)	50 (10.8)	11 (6.6)	0.128
Vasculopathy	36 (5.7)	25 (5.4)	11 (6.6)	0.563
COPD	61 (9.7)	43 (9.3)	18 (10.8)	0.647
Chronic liver failure	24 (3.8)	20 (4.3)	4 (2.4)	0.349
Chronic renal failure	63 (10)	50 (10.8)	13 (7.8)	0.295

COPD, chronic obstructive pulmonary disease; IQR, inter-quartile range; SE, status epilepticus. Values in bold indicate statistical significance, with p-values less than 0.05.

**Table 2:** First-line therapies other than benzodiazepines.

First-line non-BDZ therapy	Numbers of patients (%)
Carbamazepine	1 (0.6)
Phenobarbital	1 (0.6)
Zonisamide	1 (0.6)
Brivaracetam	2 (1.2)
Phenytoin	9 (5.4)
Lacosamide	28 (16.8)
Levetiracetam	62 (37.1)
Valproic acid	63 (37.7)

28.4% (179/630) of patients died within 30 days from SE diagnosis. The 30-day mortality rate for patients receiving BDZ as first-line therapy was 25.9% (120/463) versus 35.3% (59/167) for patients not receiving BDZ (*p* = 0.027). Table 3 summarizes the clinical variables related with 30-day mortality. Non-survivors were older, had a lower history of previous seizures and more comorbidities (ischemic heart disease, previous stroke, dementia, tumor, vasculopathy, COPD, and chronic renal failure). They were more likely to have impaired consciousness before treatment, had a higher incidence of acute hypoxic SE, and a lower incidence of remote symptomatic SE and progressive symptomatic SE. They had a higher frequency of myoclonic and non-convulsive SE, and a lower frequency of generalized convulsive SE.

**Table 3:** Clinical variables and association with 30-day mortality.

Variable	Survivors (*n* = 451)	Non-survivors (*n* = 179)	*p*-value
Patients, *n* (%)	451 (71.6)	179 (28.4)	
Sex, *n* (%)			0.587
Male	172 (38.1)	73 (40.8)	
Female	279 (61.9)	106 (59.2)	
Age, years, median (IQR)	71 (59–80)	81 (75–87)	**<0.001**
Etiological classification, *n* (%)			
Acute symptomatic, hypoxic	21 (4.7)	37 (20.7)	**<0.001**
Acute symptomatic, non-hypoxic	240 (53.2)	104 (58.1)	0.288
Remote symptomatic	94 (20.8)	13 (7.3)	**<0.001**
Progressive symptomatic	78 (17.3)	19 (10.6)	**0.038**
Other	18 (4)	6 (3.4)	0.820
SE semiology, *n* (%)			
Generalized convulsive	74 (16.4)	17 (9.5)	**0.032**
Focal motor	130 (28.8)	39 (21.8)	0.074
Non convulsive	234 (51.9)	111 (62)	**0.026**
Myoclonic	13 (2.9)	12 (6.7)	**0.039**
Prior history of epilepsy, *n* (%)	183 (40.6)	40 (22.3)	**<0.001**
Impaired consciousness before treatment, *n* (%)	104 (23.1)	95 (53.1)	**<0.001**
Comorbidities, *n* (%)			
Ischemic heart disease	42 (9.3)	34 (19)	**0.002**
Cerebrovascular disease	60 (13.3)	47 (26.3)	**<0.001**
Diabetes mellitus	83 (18.4)	44 (24.6)	0.098
Chronic heart failure	27 (8)	17 (9.5)	0.122
Dementia	68 (15.1)	42 (23.5)	**0.015**
Tumour	(35/7.8)	26 (14.5)	**0.016**
Vasculopathy	15 (3.3)	21 (11.7)	**<0.001**
COPD	32 (7.1)	29 (16.2)	**0.001**
Chronic liver failure	13 (2.9)	11 (6.1)	0.065
Chronic renal failure	28 (6.2)	35 (19.5)	**<0.001**

COPD, chronic obstructive pulmonary disease; IQR, inter-quartile range; SE, status epilepticus. Values in bold indicate statistical significance, with p-values less than 0.05.

**Figure 1:**
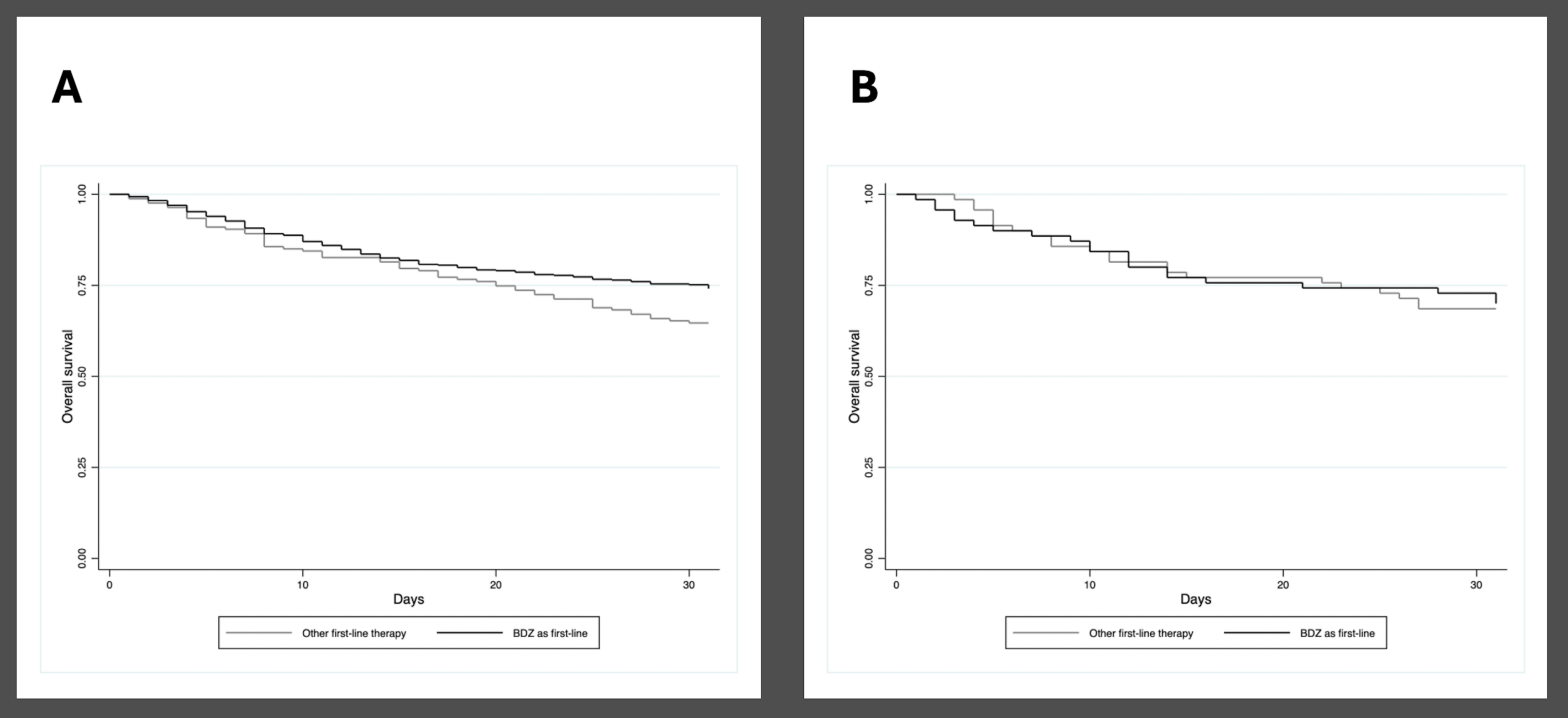
**(A)** Kaplan–Meier curve showing 30-day mortality in patients who received and did not receive benzodiazepines as first-line treatment for status epilepticus, entire study cohort. **(B)** Kaplan–Meier curve showing 30-day mortality in patients who received and did not receive benzodiazepines as first-line treatment for status epilepticus, propensity score matching sub-cohort.

Multivariate analysis adjusted for possible univariate confounders showed that using non-BDZ ASMs as a first-line therapy was not independently associated with higher 30-day mortality. According to the Kaplan–Meier analysis of 30-day mortality, BDZ-treated patients had a longer median survival (26.2; IQR 0.4) days than those who were not treated with BDZ (24.9; IQR 0.7); *p* = 0.027 (Figure 1A).

Secondary outcomes are reported in Table 4. Patients who received BDZ as a first-line treatment had less orotracheal intubation and anesthetics within 24 h of SE onset; less frequent progression to refractory and super-refractory status epilepticus; less admission to and shorter stay in intensive care units; shorter time to SE cessation, although without difference regarding achievement of SE cessation according to SENSE criteria. They had a lower rate of worsening of functional status at discharge.

**Table 4:** Occurrence of secondary outcomes among patients who received and did not receive benzodiazepines as first-line treatment for status epilepticus.

Outcomes^a^	Total	BDZ first-line therapy, 463 patients (73.5%)	Non-BDZ first-line, 167 patients (26.5%)	*p*-value
Need for anaesthetics within 24 h of SE onset, *n* (%)	134 (21.3)	76 (16.4)	58 (34.7)	**<0.001**
Progression to refractory SE, *n* (%)	171 (27.1)	101 (21.8)	70 (41.9)	**<0.001**
Progression to super-refractory SE, *n* (%)	80 (12.7)	48 (10.4)	32 (19.2)	**0.006**
Need for orotracheal intubation, *n* (%)	166 (26.4)	90 (19.4)	76 (45.8)	**<0.001**
Admission to intensive care unit, *n* (%)	210 (33.3)	124 (26.8)	86 (51.5)	**<0.001**
Length of stay in intensive care unit, days, median (IQR)	14 (8–28)	13 (7–24)	19 (10–36)	**<0.001**
CE cessation according to SENSE criteria, *n* (%)	220 (35.6)	409 (88.3)	139 (83.2)	0.107
Time to SE cessation, days, median (IQR)	2 (1–5)	2 (0–5)	3 (1–7)	**<0.001**
Worsening of functional status (mRS at discharge > pre-SE mRS), *n* (%)	374 (59.4)	253 (54.6)	121 (72.5)	**<0.001**

IQR, inter-quartile range; mRS, modified Rankin scale; SE, status epilepticus; SENSE, sustained effort network for treatment of status epilepticus.

a Outcomes are reported as number of patients (%). Values in bold indicate statistical significance, with p-values less than 0.05.

The propensity score generated a sub-cohort of 140 patients (70 treated with BDZ and 70 treated with a different drug) with overlapping baseline characteristics (Table 5). To note the propensity-matched groups are characterized by non-convulsive SE in more than 50% of the cases. In the propensity cohort of patients, the overall 30-day mortality was 30.7% (43/140), with no difference between BDZ-treated patients (30%; 21/70) and those who received a different first-line therapy (31.4%; 22/70) (*p* = 1.000). The Kaplan–Meier analysis showed no differences in survival between the two treatment groups (log rank test *p* = 0.891) (Figure 1B). Concerning secondary outcomes a shorter time to SE cessation was found among BDZ-treated patients; a non-significant trend for shorter stay in intensive care unit was also found in these patients (Table 6).

**Table 5:** Baseline characteristics of included patients, propensity score matching sub-cohort.

Variable	Total	BDZ first-line	Non-BDZ first-line	*p*-value
Patients, *n* (%)	140 (100)	70 (50)	70 (50)	
Sex, *n* (%)				0.728
Male	53 (37.9)	28 (40)	25 (35.7)	
Female	87 (62.1)	42 (60)	45 (64.3)	
Age, years, median (IQR)	71 (61–81)	70 (58–80)	75 (63–81)	0.236
Etiological classification, *n* (%)				
Acute symptomatic, hypoxic	25 (17.9)	12 (17.1)	13 (18.6)	1.000
Acute symptomatic, non-hypoxic	74 (52.9)	38 (54.3)	36 (51.4)	0.866
Remote symptomatic	16 (11.4)	7 (10)	9 (12.9)	0.791
Progressive symptomatic	21 (15)	11 (15.7)	10 (14.3)	1.000
Other	4 (2.9)	2 (2.9)	2 (2.9)	1.000
SE semiology, *n* (%)				
Generalized convulsive	22 (15.7)	12 (17.1)	10 (14.3)	0.817
Focal motor	29 (20.7)	14 (20)	15 (21.4)	1.000
Non convulsive	79 (56.4)	39 (55.7)	40 (57.1)	1.000
Myoclonic	10 (7.1)	5 (7.1)	5 (7.1)	0.061
Prior history of epilepsy, *n* (%)	55 (39.3)	27 (38.6)	28 (40)	1.000
Impaired consciousness before treatment, *n* (%)	67 (47.9)	35 (50)	32 (45.7)	0.735
Comorbidities, *n* (%)				
Ischemic heart disease	30 (21.4)	16 (22.9)	14 (20)	0.837
Cerebrovascular disease	31 (22.1)	16 (22.9)	15 (21.4)	1.000
Diabetes mellitus	31 (22.1)	15 (21.4)	16 (22.9)	1.000
Chronic heart failure	11 (7.9)	3 (4.3)	8 (11.4)	0.208
Dementia	24 (17.1)	10 (14.3)	14 (20)	0.502
Tumor	5 (3.6)	3 (4.3)	2 (2.9)	1.000
Vasculopathy	9 (6.4)	3 (4.3)	6 (8.6)	0.493
COPD	11 (7.9)	2 (2.9)	9 (12.9)	0.055
Chronic liver failure	6 (4.3)	3 (4.3)	3 (4.3)	1.000
Chronic renal failure	21 (15)	13 (18.6)	8 (11.4)	0.344

COPD, chronic obstructive pulmonary disease; IQR, inter-quartile range; SE, status epilepticus.

**Table 6:** Occurrence of secondary outcomes among patients who received and did not receive benzodiazepines as first-line treatment for status epilepticus, propensity score matching sub-cohort.

Outcomes^a^	Total	BDZ first-line therapy, 70 patients (50%)	Non-BDZ first-line, 70 patients (50%)	*p*-value
Need for anesthetics within 24 h of SE onset, *n* (%)	42 (30)	18 (25.7)	24 (34.3)	0.357
Progression to refractory SE, *n* (%)	53 (37.8)	23 (32.9)	30 (42.9)	0.296
Progression to super-refractory SE, *n* (%)	29 (20.7)	14 (20)	15 (21.4)	1.000
Need for orotracheal intubation, *n* (%)	57 (40.7)	25 (35.7)	32 (46.4)	0.230
Admission to intensive care unit, *n* (%)	66 (47.1)	28 (40)	38 (54.3)	0.127
Length of stay in intensive care unit, days, median (IQR)	16 (8–36)	12 (6–26)	20 (9–41)	**0.049**
Return to pre-SE condition at discharge, *n* (%)	44 (31.4)	20 (28.6)	24 (34.3)	0.585
CE cessation according to SENSE criteria, *n* (%)	93 (66.4)	45 (64.3)	48 (68.6)	0.721
Time to SE cessation, days, median (IQR)	3 (1–7)	1 (0–4)	4 (2–8)	**<0.001**
Worsening of functional status (mRS at discharge > pre-SE mRS), *n* (%)	91 (65)	43 (61.4)	48 (68.6)	0.479

IQR, inter-quartile range; mRS, modified Rankin scale; SE, status epilepticus; SENSE, sustained effort network for treatment of status epilepticus.

a Outcomes are reported as number of patients (%).

## Discussion

In our 9-years cohorts the use of non-BDZ drugs as first line SE treatment was about 25%. This figure of real word practice underscores a discrepancy between clinical practice guidelines and real word SE management and is in line with previous reports (15–17, 25). Indeed, although the efficacy of BDZ has been confirmed in RCTs and no other medicine has proven to be more effective, medications other than BDZ are frequently used as first agents in real-world practice. In over 20% of the patients in the SENSE registry, the first-line treatment was with antiseizure medications different from BDZ (mostly levetiracetam) (25). Similarly, the STEPPER study conducted in Italy reported that non-BDZ ASMs were used as first-line agents in 29% of SE episodes (15), whereas in a global audit of treatments of refractory SE, only 33% of cases (156/474 patients) received BDZ as first-line drugs (26).

Because BDZs are considered the first-line treatment for SE, they have been used to compare the efficacy of other therapeutic options in clinical trials (27, 28). A first RCT comparing i.v. diazepam to phenytoin, lorazepam, phenobarbital, and phenytoin found that lorazepam was more efficacious than phenytoin. Even though there was no difference in efficacy between lorazepam and phenobarbital or diazepam and phenytoin, the authors felt that it was easier to utilize (27). A second RCT showed that adding levetiracetam to clonazepam had no advantage over clonazepam alone in the treatment of generalized convulsive seizures in the prehospital setting (28).

In our study, the analysis of outcomes data according to first-line used drugs in the whole cohort of patients showed that patients who received BDZ had less orotracheal intubation and anesthetics within 24 h of SE onset; less frequent progression to refractory and super-refractory status epilepticus; less admission to and shorter stay in intensive care units; shorter time to SE cessation, and a lower rate of worsening of functional status at discharge. Therefore, even if patients who received BDZ as first-line treatment were unbalanced respect to several clinical variable compared to patients who received other ASMs, these results should alert us to the use of non-benzodiazepines as first-line drugs, as supported by clinical practice guidelines (6–8).

Because these medicines are often highly lipophilic, they can pass the blood–brain barrier rapidly to reach their neuronal targets, explaining their clinical efficacy and rapid onset of action. The evidence supporting their use as first-line treatment for SE is substantiated by results of several RCTs, confirming their efficacy in achieving SE cessation (11–14). Owing to the intrinsic severity of this condition, only three RCTs used placebo as a comparator to evaluate the efficacy of BDZs in patients with “premonitory” SE, described as acute repetitive seizures (11, 29–31). They established the efficacy and safety of these drugs, showing that intravenous or intrarectal diazepam and intravenous lorazepam are more effective than placebo in reducing the risk of SE continuation, with a lower need for ventilatory support or use of a different drug or general anesthesia to achieve SE cessation. These findings provided clinical evidence for BDZs as the gold standard for the initial treatment of SE.

Here, we also provided the evidence that at least in a subpopulation of SE patients the use of first-line ASMs instead of BDZ has negligible effects on measures of outcomes. Indeed, after balancing the clinical characteristics that differed between first-line therapies with BDZ or with a different drug using propensity score matching, no difference in 30-day mortality was detected. Among the other secondary outcome measures, only time to SE cessation was shorter in BDZ-treated patients, while all other secondary outcomes measures were similar in the two groups.

In other terms, the use of a BDZ or of a drug with different antiseizure property might not be the main prognostic factor in SE patients. These results, however, should be interpreted with caution, as the generalizability of our results is affected by the characteristics of the sub-cohort obtained through propensity score matching, which may not be considered as representative of the whole SE patient population. Interestingly, in this sub-cohort more than half of the patients had non-convulsive SE (56.4%), whereas only a few had generalized convulsive SE (15.7%). These characteristics could influence the decision to use BDZs as first-line treatment and could affect the prognosis about mortality and secondary outcomes.

The impact of adherence to treatment guidelines on mortality and functional prognosis in SE remains a subject of ongoing debate. While some studies indicate a significant association, others suggest a more limited prognostic influence. For instance, a prospective single-center study conducted in Switzerland found that improved adherence to SE treatment guidelines had no significant effect on mortality and functional outcomes (18). Conversely, a recent systematic review encompassing 22 studies published between 1970 and 2018 reported that nonadherence to SE management guidelines was associated with increased risk of adverse outcomes, including admission to the intensive care unit and mortality (20).

Based on our findings, adherence to guideline-recommended use of BDZ as initial therapy may have limited impact on prognosis in certain patient subgroups (particularly those with non-convulsive SE), emphasizing the role biological factors such as etiology and intrinsic SE severity (18). At the same time, our results pave the way for future research aimed at evaluating the role of other drugs that could be used as valid alternatives to BDZ as first-line agents for SE. Although i.v. lorazepam or intramuscular midazolam effectively control early SE in approximately 63–73% of cases, more effective drugs that can achieve SE cessation in a larger proportion of patients are still needed. Furthermore, BDZ have a short antiseizure activity due to their high lipophilicity, resulting in rapid redistribution to peripheral adipose tissue. This explains why, after 2 h of successful treatment with diazepam, SE relapses in more than half of patients (32). There remains, hence, the need to prolong the anticonvulsant effect of BDZ without increasing the risk of adverse effects. Unfortunately, due to the heterogeneity of drugs other than BDZ used as first line therapies in our study, we cannot draw any conclusion on the efficacy and safety of individual medications. However, taken as a whole, the use of antiseizure medications might represent a meaningful alternative to the use of BDZ, at least in non-convulsive SE. Indeed, in suspected non-convulsive SE, these drugs could prove useful both for treatment and for diagnostic confirmation and have been proposed as a rationale alternative, particularly for assessing treatment response without inducing the sedative effects commonly associated with BDZ use (33). Also, in minimally symptomatic or asymptomatic non-convulsive SE detected through routine EEG, non-BDZ agents such as levetiracetam or valproate may be preferred over BDZs due to reduced sedative burden, particularly when treatment initiation is delayed, or clinical signs are minimal. While BDZs are effective first-line treatments for convulsive status epilepticus due to their rapid onset, their efficacy in non-convulsive SE—especially when symptoms are subtle or absent—may be limited, potentially due to delayed recognition and treatment.

On the other hand, considering the high mortality and morbidity associated with SE, and the lack of reliable biomarkers to identify patients who do not respond to BDZ, a more aggressive initial treatment could be considered in selected cases to promptly interrupt the ongoing epileptic activity. This could potentially involve earlier anesthetic treatment, which has recently been correlated with shorter median SE duration and higher returns to premorbid neurologic function (34).

### Study limitation

This study has some limitations. This study was conducted in a single tertiary care center, which may restrict the findings’ generalizability. Additional research in diverse cohorts and hospital settings is required to confirm our findings. The study’s primary strengths are the large sample size and the use of propensity score matching, a statistical sampling technique that can limit the selection bias by adequately matching 1 to 1 to achieve a balance in prognostic characteristics at baseline. This statistical approach mimics some features of a RCT and reduces confounding by indication, although it cannot eliminate all biases and weaknesses of observational studies. Although matching is not possible for unknown prognostic factors or variables that have not been collected, we paid special attention to include all possibly relevant prognostic variables in the analysis. However, the propensity-score matching sub-cohort primarily included non-convulsive SE and only a small fraction of generalized convulsive SE, which clearly limit the generalizability of our findings to non-convulsive SE. Furthermore, in this study we included all types of SE due to a wide variety of etiologies, including post-anoxic SE. However, performing analyses on each SE type or on specific etiologies would have reduced the number of patients, decreasing and potentially hampering the informative potential of the study. Larger studies leading to larger propensity score sub-cohorts are hence required to confirm our results and to evaluate whether BDZ given as a first-line treatment for SE affect 30-day mortality and other outcomes in specific SE types or etiologies (35).

## Conclusion

The use of non-BDZ first-line treatment was found to be frequent, approaching 25%. The impact of these deviations from clinical practice guidelines was associated with worse outcome measures in the primary analysis. However, our propensity-core matching analysis shows that in some patients, mainly with non-convulsive SE, the overall prognosis of SE might only be partially dependent on the first medications administered and could be more influenced by other biological variables (36). Our findings pave the way for future research into additional therapies for this serious condition.

## Data Availability

The datasets presented in this article are not readily available because original data will be shared upon reasonable request to the corresponding author. Requests to access the datasets should be directed to stefano.meletti@unimore.it.
