# Supplementary material for: Benzodiazepines versus non-benzodiazepine antiseizure medications as first-line agents for status epilepticus: analysis of real word data from a 9-years prospective cohort
Source: Front Neurol. 2025 Nov 12;16:1681483. doi: 10.3389/fneur.2025.1681483 (PMC12649711; doi:10.3389/fneur.2025.1681483)
Supplement: Supplementary file 1 [file Table_1.DOCX]

**Supplementary material:**

- Internal protocol for the pharmacological treatment of status epilepticus, summarized and translated from: Percorso Diagnostico Terapeutico Assistenziale per la diagnosi e il trattamento dello Stato Epilettico dell’adulto (Diagnostic-therapeutic care path for the diagnosis and treatment of status epilepticus in adults), StEp

**INTERNAL PROTOCOL FOR THE PHARMACOLOGICAL TREATMENT OF STATUS EPILEPTICUS**

**Summarized and translated from: Percorso Diagnostico Terapeutico Assistenziale per la diagnosi e il trattamento dello Stato Epilettico dell’adulto (Diagnostic-therapeutic care path for the diagnosis and treatment of status epilepticus in adults), StEp**

This treatment protocol was adapted from: Brophy GM, Bell R, Claassen J, Alldredge B, Bleck TP, Glauser T, Laroche SM, Riviello JJ Jr, Shutter L, Sperling MR, Treiman DM, Vespa PM; Neurocritical Care Society Status Epilepticus Guideline Writing Committee. Guidelines for the evaluation and management of status epilepticus. Neurocrit Care. 2012 Aug;17(1):3-23.

***FIRST-LINE TREATMENTS:***

LORAZEPAM IV: 0.1 mg/kg up to a dose of 4 mg, diluted in 10 cc of physiological solution, administered slowly, at a maximum rate of 2 mg/min. Repeatable once (after at least 5-10 minutes) up to a maximum dose of 8 mg.

DIAZEPAM IV: 0.15 mg/kg up to a dose of 10 mg, diluted in 10 cc of physiological solution, administered slowly, at a maximum rate of 5 mg/min. Repeatable once (after at least 5 min) up to the maximum dose of 20 mg.

**In case of failure to find venous access**

MIDAZOLAM im: 0.2 mg/Kg up to a maximum dose of 10 mg (5 mg if weight < 40Kg or if elderly).

DIAZEPAM er: 0.2 mg/Kg (if > 12 years)

***SECOND-LINE TREATMENTS:***

In patients who already have received adequate doses of benzodiazepines:

Phenytoin: 20 mg/kg i.v. at max 50 mg/min

**Possible alternatives:**

Sodium valproate: 20-40 mg/Kg at max 3-6 mg/Kg/min

Levetiracetam 20-60 mg/kg (max 3000 mg) at max 2-5 mg/kg/min

Lacosamide 200-400 mg (200 mg in 15 min)

***TREATMENT OF REFRACTORY STATUS EPILEPTICUS***

- propofol: loading dose: 1-2 mg/kg

Maintenance dose: 30-200 ug/Kg/min*

- midazolam: loading dose: 0.2 mg/kg at infusion rate of 2 mg/min

Maintenance dose: 0.05 – 2 mg/Kg/h*

- thiopental: loading dose: 2-7 mg/kg at infusion rate ≤ 50 mg/min

Maintenance dose: 0.5-5 mg/Kg/h*

* maintenance dose should be established based on EEG monitoring.
